# Is there a role for consolidative radiotherapy in the treatment of aggressive and localized Non-Hodgkin Lymphoma? A systematic review with meta-analysis

**DOI:** 10.1186/1471-2407-12-288

**Published:** 2012-07-13

**Authors:** Lucas Vieira dos Santos, JoãoPaulodaSilveiraNogueira Lima, Carmen Sílvia Passos Lima, Emma Chen Sasse, André Deeke Sasse

**Affiliations:** 1 Departamento de Oncologia Clínica, Hospital de Câncer de Barretos, Barretos, Brazil; 2 CEVON – Centro de Evidências em oncologia, Universidade Estadual de Campinas, UNICAMP, Campinas, Brazil; 3 Departamento de Clínica Médica, Centro de Evidências em Oncologia – CEVON, Faculdade de Ciências Médicas, Universidade Estadual de Campinas – UNICAMP, Campinas, SP CEP 13083-970, Brazil

**Keywords:** Non-Hodgkin lymphoma, Consolidative radiotherapy, Meta-analysis, Systematic review

## Abstract

**Background:**

Chemotherapy is the mainstay of non-Hodgkin lymphoma (NHL) treatment. Based on expert opinion, the use of radiotherapy (RT) is currently preferred in some institutions as consolidative treatment for patients with localized disease. The lack of conclusive data coming from conflicting studies about the impact of treatment demands a systematic review, which could provide the most reliable assessment for clinical decision-making. We evaluate the addition of RT post-CT, for aggressive and localized NHL (ALNHL).

**Methods:**

Randomized controlled trials (RCT) that evaluated chemotherapy alone versus chemotherapy plus RT were searched in databases. The outcomes were overall survival (OS), progression-free survival (PFS), overall response rate (ORR) and toxicity. Risk ratio (RR) and hazard ratio (HR) with their respective 95% confidence intervals (CI) were calculated using a fized-effect model.

**Results:**

Four trials (1,796 patients) met the inclusion criteria. All trials tested the use of RT after systemic therapy comprising anthracycline-based chemotherapy. This systematic review showed that RT enhances PFS after chemotherapy (hazard ratio [HR] 0.81; 95% CI 0.67-0.98; p = 0.03), with no impact on ORR and OS. Some heterogeneity between trials could limit the conclusions about OS. Toxicity data could not be pooled due to differences in reporting adverse events.

**Conclusions:**

This systematic review with meta-analysis shows no improvement in survival when adding RT to systemic therapy for ALNHL. Our conclusions are limited by the available data. Further evaluations of new RT technologies and its association with biologic agents are needed.

## Background

Non-Hodgkin lymphoma (NHL) is the sixth most common cause of cancer death in the world [1]. About 300,000 new cases of NHL occur every year, accounting for nearly 3% of all new cases of cancer. Among them, more than half will die due to NHL [2].

There are several classifications currently in use for NHL that are somewhat overlapping. The Revised European American Lymphoma (REAL) classification [3] was initially proposed. However, a REAL-based classification is now widely accepted, also known as the World Health Organization (WHO) classification for hematologic malignancies. This classification is based on cell of origin, maturity, morphology, immunophenotype, genetic and clinical features [4]. Considered outdated by many, the International Working Formulation [5] (WF) had divided NHL in low, intermediate and high grade, based on morphology and natural history. It remains important because studies designed more than 10 years ago were based on this classification.

In some series, more than one third of patients with NHL have an aggressive phenotype. For patients with localized disease, radiotherapy (RT) was the first curative approach and continues to be a part of combined modality therapy [6-8]. Previous studies have shown that almost 70% of patients with localized NHL can be cured by RT alone [9,10]. However, the high relapse rate outside the radiation field justifies the requirement of chemotherapy in this setting [11,12].

Since the early 1980s, the synergistic effect of both modalities has been evaluated, and their combination widely advocated in the treatment of patients with NHL [13-17]. The benefit of this strategy was first shown in a randomized trial including 316 patients, comparing RT versus chemotherapy versus combined therapy [18]. In this trial, the combination arm had better failure-free survival and OS when compared to chemotherapy alone, or RT alone arms. The use of RT after systemic therapy is frequently recommended, based on expert opinion, to improve local control of the disease, and possibly to diminish relapse and death [19].

The lack of conclusive data coming from conflicting studies about the impact of treatment demands a systematic review, which could provide the most reliable assessment for clinical decision-making. The aim of this systematic review is to assess whether adding RT to standard chemotherapy for aggressive and localized NHL (ALNHL) has an impact on local tumor control and survival.

## Methods

This study was approved by the Ethics Committee at Universidade Estadual de Campinas.

### Types of studies

Randomized controlled trials (RCT) were included comparing chemotherapy and consolidative RT versus chemotherapy alone for patients with ALNHL.

### Types of participants

Participants included adults with ALNHL, without previous treatment. We defined localized disease as Ann Arbor stage I and stage II with contiguous disease encompassed by a radiation field [20]. We defined aggressive NHL according to the WF [5], the REAL classification [3], or the WHO classification [4]. In older trials, an intermediate or a high-grade classification was permitted.

### Types of outcome measures

The primary outcome was OS. Secondary outcomes of interest were PFS, response to therapy and safety. PFS encompassed the term disease-free survival (DFS), used when there was no residual disease.

### Search strategies for identification of studies

We performed an electronic database search [21] in MEDLINE, EMBASE, Cochrane Central Register of Controlled Trials – CENTRAL and LILACS (until January 2010); electronic or hand searching of the conference proceedings between 1980 and 2009 of the American Society of Clinical Oncology (ASCO), the American Society for Therapeutic Radiology and Oncology (ASTRO), the American Society of Hematology (ASH), the European Conference on Clinical Oncology (ECCO) and the European Society of Medical Oncology (ESMO). Reference list of all recovered trials and relevant reviews were also considered. For electronic databases, we used a sensitive search strategy with words linked to NHL and RT.

### Trial selection

Titles and abstracts of studies identified from search strategy were screened independently by two reviewers (LVS and JPL), according to the eligibility criteria described above. Disagreements in the trial selection were resolved by discussion and a third reviewer was invited to give his opinion (ADS) if consensus was not reached. Full-text versions of all eligible studies were obtained for quality assessment and data extraction.

### Quality assessment

The quality of each individual study was assessed independently by two reviewers (LVS and ADS) using the published manuscript. A specific data extraction form was designed for assessment of quality features of studies, such as randomization, allocation concealment, intention-to-treat principle, similarity of treatment arms according to known prognostic factors, follow up and drop-outs [22,23]. Disagreements were discussed among the group until consensus was reached.

### Data extraction

A data extraction form was designed previously, and included the following items: general identification information (authors, title, journal, date of publication, protocol name, and duplication of publication), trial, type of patients, intervention characteristics, and reported outcomes. Data extraction was performed independently by two reviewers (LVS and JPL) and disagreements were resolved by consensus. When it was not possible to obtain data from the published trial, we tried to contact the authors to provide the information or additional data.

Data were directly extracted from the published data, or estimated from survival curves using the methods described by Parmar and colleagues [24]. Calculations were carried out using the spreadsheet provided by Tierney and colleagues [25].

### Statistical analysis and synthesis

Details regarding the main methodological dimensions empirically linked to bias as described by Deeks and colleagues [26] were extracted and the methodological quality of each selected trial was assessed by two reviewers (LVS and ADS). Special attention was given to the generation and concealment of the sequence of randomization, whether an intention-to-treat analysis was performed or not, and source of funding. These data were applied in sensitivity analyses to test the stability of the results.

Review Manager 5 software (RevMan 5; The Nordic Cochrane Centre, The Cochrane Collaboration, Copenhagen, Denmark) was used to perform the meta-analysis. For time-to-event variables, the effect of the treatment for each single study was expressed as a hazard ratio (HR) of chemotherapy plus RT arm over the chemotherapy alone arm. The 95% confidence interval (CI) was calculated for each point estimate. For dichotomous variables, the effect of treatment was calculated as a risk ratio (RR), and presented with the correspondent 95% CI. Data were analyzed using Mantel-Haenszel fixed-effect method.

Statistical heterogeneity of the results of the trials was assessed by the chi-square test [27], expressed with the I^*2*^ index, as described by Higgins and colleagues [28]. When heterogeneity was detected, a possible explanation for it was intensively pursued. If a reasonable cause was found, a separate analysis was then performed. When the cause was not apparent and heterogeneity was caused by divergent data in terms of direction of results, we chose not to pool the data. Publication bias was evaluated by the Egger test [29,30].

## Results

### Description of studies and quality assessment

**Figure 1:**
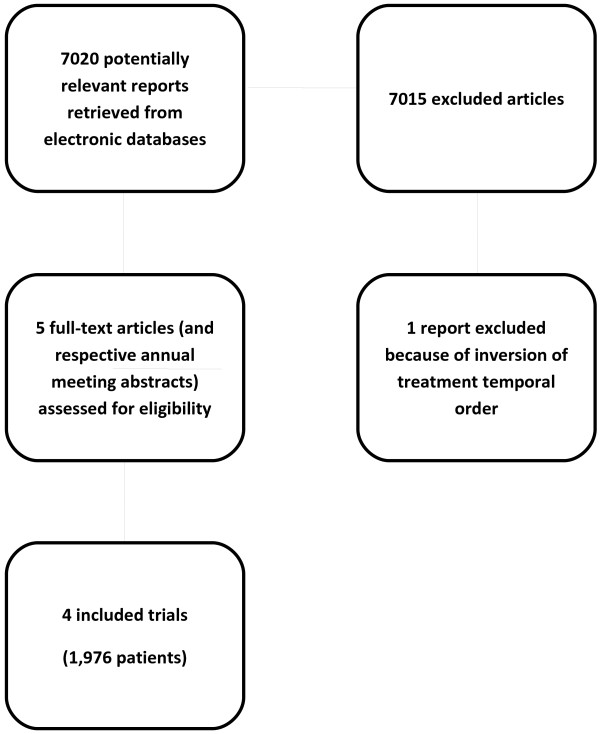
Flow diagram through the different phases of the review.

Four trials with a total of 1,796 patients met the inclusion criteria (Figure 1) [31-35]. One study was excluded because RT was employed before chemotherapy in the combined modality arm [18]. All data were extracted from the original peer-reviewed publication. There was no evidence of publication bias (Egger’s test: p = 0.52). The methodological characteristics of the selected trials included in this meta-analysis (Table 1) had no impact on the results obtained, as confirmed by the sensitivity analysis performed [this data is available upon request].

**Table 1:** Main characteristics of selected trials related to biasTrial

	**Year of Publication**	**Random Assignment**	**Allocation Concealment**	**Withdrawn Description**	**α-error**	**β-error**	**ITT**	**Site**	**Sponsor**
**SWOG 8736**	1998	Adequate	Unclear	Yes	No	No	Yes	Multicentric	Public
**ECOG 1484**	2004	Adequate	Adequate	Yes	Yes	Yes	Yes	Multicentric	Public
**GELA LNH 93-1**	2005	Adequate	Adequate	No	Yes	Yes	Yes	Multicentric	Both
**GELA LNH 93-4**	2007	Adequate	Adequate	No	Yes	Yes	Yes	Multicentric	Both

All trials tested the use of RT after systemic therapy comprising anthracycline-based chemotherapy (Table 2). Treatment with cyclophosphamide, doxorubicin, vincristine and prednisone (CHOP) was the preferred regimen in these trials.

**Table 2:** Study population and therapy

**Trial**	**Arms**	**N**	**Population**	**Age and PS 0-1**	**Stage**	**LDH**	**IPI**	**RT**
**SWOG 8736**	CHOP x 8	201	WF groups D-J (DLBCL 75%); stage I to II non-bulky	> 60y = 49% 0-1 = 96%	II 33%	↑ 19%	0-1 = 71%	No RT
	CHOP x 3 + RT	200		>60y = 50% 0-1 = 97%	II 32%	↑ 20%	0-1 = 74%	IFRT 40-55 Gy
**ECOG 1484**	CHOP x 8	112	WF H-J (DLBCL 83%); stage I bulky to II; complete responders to CHOP only	Median 60y 0-1 = 93%	II 70% E 52%, B20%	NA	NR	No RT
	CHOP x 8 + RT	103		Median 58y 0-1 = 96%	II 68% E 45% B22%	NA	NR	IFRT 30 Gy
**GELA LNH 93-1**	ACVBP + CT	318	WF H-J or anaplastic according to uKC (DLBCL 81%); 15-60y; aaIPI = 0†; stages I to II	Median 46y 0-1 = 100%	II 32% E 46%	↑ 3%	0 = 96%†	No RT
	CHOP x 3 + RT	329		Median 47y 0-1 = 100%	II 32% E 52%	↑ 3%	0 = 95%†	IFRT 40 Gy
**GELA LNH 93-4**	CHOP x 4	277	WF H-J or anaplastic according to uKC (DLBCL 80%); >60y; aaIPI = 0†; stages I to II	Median 69y 0-1 = 99%	II 32% Bu 8%	↑ 2%	0 = 95%†	No RT
	CHOP x 4 + RT	299		Median 68y 0-1 = 99%	II 34% Bu 9%	↑ 3%	0 = 95%†	IFRT 40 Gy

**Abbreviations**: ACVBP + CT: doxorubicin 75 mg/m² D1 + cyclophosphamide 1200 mg/m² D1 + vindesine 2 mg/m² D1,5 + bleomycin 10 mg D1,5 and prednisone 60 mg/m² D1-5, 3 cycles with 14 days each and then consolidative chemotherapy with methotrexate, folinic acid, etoposide, ifosfamide and cytarabine; B: “B” symptoms; Bu: bulky disease; CHOP: cyclophosphamide 750 mg/m² D1 + doxorubicin 50 mg/m² D1 + vincristine 1,4 mg/m² D1 + prednisone 60 mg/m² or 100 mg D1-5, 21 day cycles; DLBCL: diffuse large B cell lymphoma; E: extranodal disease; IFRT: involved-field radiotherapy; IPI: International Prognostic Index; LDH: lactate dehydrogenase; NA: not available; NR: not reported; uKC: updated Kiel Classification; WF: Working Formulation; † age-adjusted IPI.

The Southwest Oncology Group (SWOG) 8736 trial [32] included 401 patients with intermediate- or high-grade NHL. Stages I and II were allowed, except those with stage II and bulky disease. Participants were randomized to receive 8 cycles of CHOP or 3 cycles of CHOP plus involved-field radiation therapy (IFRT) with doses of 40 to 55 Gy. The trial was designed to address if the addition of RT may allow the use of less cycles of chemotherapy. Approximately 20% of the patients had two or more risk factors (international prognostic index [IPI] ≥ 2) [36]. Complete response occurred in 73% of chemotherapy patients, and in 75% of chemotherapy plus RT patients. With a median follow-up of 4.4 years, five-year PFS was 64% for CHOP x 8, and 77% for CHOP x 3 plus RT (p = 0.03). Five-year OS was 72% and 82% (p = 0.02), respectively. In an updated analysis, the results showed an excess of late relapses and deaths due to lymphoma in the combined-modality treatment. PFS and OS curves of the two arms overlapped at 7 and 9 years, respectively [33]. Unfortunately, it was impossible to use these updated results in meta-analysis, due to lack of extractable data.

The ECOG 1484 trial [34] included patients with a less favorable prognosis. All patients had at least bulky or extranodal disease, most of them were in stage II, and only high-grade NHL was allowed. This study included 352 patients receiving 8 cycles of CHOP. Those who achieved complete response (61%) were randomized to receive involved field radiation treatment (IFRT) with doses of 30 Gy (n = 103) or observation (n = 112). There were imbalances between groups, with patients allocated to receive RT presenting more mediastinal involvement and bulky disease. Six-year DFS was 53% for observation and 69% for RT (p = .05), and six-year OS rates were 67% and 79% (p = .23), respectively. The trial was designed to detect a 20% improvement in two-year DFS using a one-sided significance test. Thus, the sample size was not adequate to detect a survival advantage of consolidative RT.

In the Groupe d'Etudes des Lymphomes de l'Adulte (GELA) LNH 93–1 trial [31], 647 patients with ALNHL under 60 years and without age-adjusted IPI risk factors [36] (ECOG-PS < 2 and normal lactate dehydrogenase [LDH]) were randomized to receive an intensified induction chemotherapy (ACVBP) and a consolidative chemotherapy regimen or 3 cycles of CHOP plus 40 Gy IFRT. Bulky disease was present in 11% of patients and extranodal involvement in 50%. Complete response rate was 93% for chemotherapy alone and 92% for combined modality treatment. Five-year PFS was 82% for ACVBP versus 74% for CHOP plus RT (p < 0.001). Five-year OS was 90% and 81%, respectively (p = 0.001).

The GELA LNH 93–4 trial [35] randomized 576 patients older than 60 with ALNHL and without age-adjusted IPI risk factors [36] to receive 4 cycles of CHOP alone or 4 cycles of CHOP plus 40 Gy IFRT. Bulky disease was present in 9% of patients and extranodal involvement in 48%. Accrual was interrupted earlier due to increasing evidence that rituximab could improve efficacy in patients with aggressive NHL [37,38]. With a median follow-up of 7 years, 5-year PFS was 61% for CHOP alone and 64% for CHOP plus RT (p = 0.56), and 5-year OS was 72% and 68%, respectively (p = 0.54).

### Effects of interventions

#### Response rate

**Figure 2:**
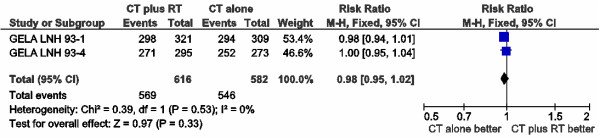
Meta-analysis of overall response rate.

**Figure 3:**
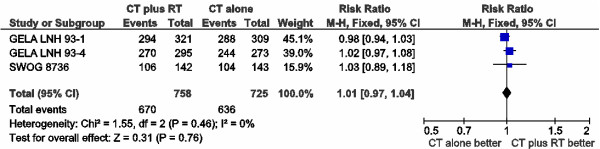
Meta-analysis of complete response rate.

There were divergences in the definition of response assessments across, but not within trials. Unconfirmed complete response was analyzed as complete response in GELA trials [31,35]. Only complete responders could be randomized to receive RT or observation in the ECOG 1484 trial [34]. SWOG 8736 [32,33] described only complete response, so data could not be used for overall response rate evaluation. Data from 1,198 patients were available. Response was analyzed according to assessable patients, as reported in these RCT. Overall response rates (ORR) were not different between groups (RR 0.98; 95% CI 0.95-1.02; p = 0.33), with no heterogeneity between trials (p = 0.53; I² = 0%) (Figure 2). Data from 1,483 patients could be pooled for complete response rate (RR 1.01; 95% CI 0.97-1.04; p = 0.76), with no heterogeneity among trials (p = 0.46; I² = 0%) (Figure 3).

#### Progression-free survival

**Figure 4:**
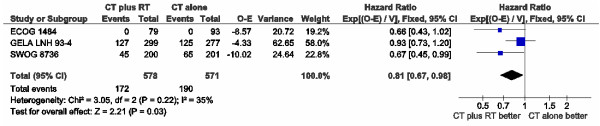
Meta-analysis of progression-free survival after exclusion of GELA LNH 93-1.

Four trials comprising 1,796 patients were analyzed for PFS [31,32,34,35]. Data from the ECOG 1484 trial [34] could not be obtained for an intention-to-treat analysis due to insufficient data. Thus, estimation was made from the published disease-free survival curve from per-protocol patients. Data from all four trials could not be pooled due to heterogeneity among them (p = 0.0001; I² = 86%). The heterogeneity persisted even excluding trials without intention-to-treat data (p = 0.0002; I² = 88%) [34]. The GELA LNH 93–1 trial [31] seemed to be the main cause of heterogeneity, maybe due to considerable differences in intensity and duration of systemic therapy in both arms. Excluding the GELA LNH 93–1 trial [31], PFS was longer for combined-modality treatment (HR 0.81; 95% CI 0.67-0.98; p = 0.03), with an acceptable heterogeneity among trials (p = 0.03; I² = 35%) (Figure 4).

Pooling data from trials whose arms had the same chemotherapy regimen (791 patients) [34,35] showed that the addition of RT to systemic therapy resulted in no improvement in PFS (HR 0.86; 95% CI 0.69-1.06; p = 0.16). In this analysis, it was not possible to exclude heterogeneity among trials (p = 0.17; I² = 46%).

#### Overall survival

Again, data from ECOG 1484 trial [34] could not be obtained for an intention-to-treat analysis due to lack of an adequate description of results. Thus, estimation was made from the published overall survival (OS) curve from “as treated” patients. Although all trials [31,32,34,35] reported data of survival analysis comprising 1,796 patients, results from four trials could not be pooled due to heterogeneity among them (p = 0.0009; I² = 82%). Even excluding the ECOG 1484 trial, there was still heterogeneity detected among trials (p = 0.0009; I² = 86%). The GELA LNH 93–1 trial [31] seemed to be mainly responsible for the heterogeneity, again, possibly due to considerable differences in intensity and duration between systemic therapy in both arms. However, even excluding the GELA LNH 93–1 trial [31], we could still detect heterogeneity among trials (p = 0.04; I² = 68%).

Pooling data only from the studies whose arms had the same chemotherapy regimen [34,35], the addition of RT showed no improvement in OS (HR 1.00; 95% CI 0.79-1.26; p = 0.99). Even in this analysis, there was considerable heterogeneity among trials (p = 0.15; I² = 51%).

#### Toxicity

In the SWOG 8736 trial [32] there were no differences in life-threatening toxicity or death between chemotherapy alone and chemotherapy plus RT arms (30% and 40%; p = 0.06; one death in each arm). Patients assigned to chemotherapy alone arm had more congestive heart failure than patients assigned to chemotherapy plus RT arm. More patients in the chemotherapy alone arm did not complete the assigned regimen (28 and 3 patients, respectively, p < 0.01).

In the ECOG 1484 trial [34], toxicity was described according to the phase of therapy (chemotherapy and then RT or observation). There was only one grade 4 adverse event (thrombocytopenia) in the RT arm and none in the observation arm. GELA LNH 93-1 [31] and 93-4 [35] did not describe toxicity individually for each arm, but life-threatening toxicity appeared to occur in a small number of patients.

Due to differences in reporting adverse events, it was not feasible to pool toxicity data. Radiation therapy appeared to be well tolerated, with side effects dependent mainly on the location of disease.

#### Risk of bias

There was no evidence of publication bias (Egger’s test: p = 0.52). The methodological characteristics of the selected trials included in this meta-analysis (described in Table 1) had no impact on the results obtained, as confirmed by the sensitivity analysis performed [this data is available upon request].

## Discussion

Early stage aggressive NHL is a heterogeneous group of diseases with a high potential of cure. Systemic therapy has been the mainstream of therapy for more than 20 years. Additionally, RT had shown to increase regional control, which may lead to improvement in survival. Other possible secondary benefits of radiotherapy could be the diminishing of chemotherapy late effects.

Considering that relapses usually occur at the site of disease, and RT alone can cure up to 70% of patients, it seems rational to test RT after chemotherapy in patients with ALNHL. The achievement of local control could be, in this setting, a surrogate of survival improvement.

Since the publication of the SWOG 8736 trial [32], radiation therapy has become the standard therapy in North America. Unfortunately, the benefit of the treatment in prolonging PFS and OS has not been sustained over the years [33]. The ECOG 1484 trial [34] evaluated the addition of RT in patients with complete response with unfavorable results. In the trial, DFS (but not OS) was better in the combination arm. The GELA LNH 93–1 trial [31] compared short-course chemotherapy regimen plus RT to an intensified chemotherapy regimen. The group receiving the ACVBP regimen showed improved survival when compared to the group receiving CHOP for 8 cycles [39] for non-localized NHL or CHOP plus RT for localized disease [31]. Despite that, the more intensive regimen has not been widely accepted due to long term adverse events, like acute myelogenous leukemia, myelodysplastic syndrome and lung cancer [40]. This is especially important for patients with one IPI risk factor [36], in whom cure rates usually exceed 80% with CHOP-based chemotherapy [31,32]. Thus, some advocate ACVBP should not be considered a valid alternative to CHOP in patients without high risk of recurrence. Recently, ACVBP plus rituximab (R-ACVBP) was compared to CHOP plus rituximab in young patients with advanced diffuse large B-cell lymphoma. With a median follow up of 44 months, R-ACVBP improved overall survival [41]. Long term follow up is needed to address long term adverse events.

Both the GELA LNH 93-1 [31] and SWOG 8736 [32,33] trials suggested that radiotherapy cannot replace inadequate chemotherapy regimens. The GELA LNH 93–4 trial [35] evaluated the addition of RT to 4 cycles of CHOP in the treatment of elderly patients with ALNHL. No improvements in terms of PFS or OS were observed. Five-year PFS in the chemotherapy alone arm was 61%, comparable to 64% in the chemotherapy alone arm after 8 cycles of CHOP in the SWOG trial in younger patients with a more favorable prognosis [32].

This systematic review showed that RT could enhance PFS after chemotherapy, with no impact on ORR and OS. Heterogeneity among trials limits a definite conclusion. The interventions under study and trial design characteristics, such as definition of outcomes, inclusion criteria, risk factors and statistical considerations can cause substantial differences when pooling data, and may constitute the major causes of heterogeneity found in the current meta-analysis. Our results are consistent with those addressed in an overview published in 2003 [7]. The high efficacy of chemotherapy in inducing remission of ALNHL would increase the number of subjects in clinical trials evaluating the role of consolidative RT. Thus, all included trials were individually underpowered to assess improvement of response rate, PFS and OS.

More recently, rituximab has been incorporated into the treatment of patients with NHL, with clear benefits in PFS and OS [37,38]. Now the combination of rituximab with a CHOP-based chemotherapy regimen has been considered as the standard treatment for aggressive NHL patients. There is still no prospective trial evaluating the role of RT in the era of targeted therapy, so far the role of radiation after chemotherapy plus rituximab remains speculative. Recently, a retrospective study suggested improvement in both OS and PFS with consolidative RT after rituximab-CHOP chemotherapy. This improvement occurred in both early (I and II) and advanced stage (III and IV) [42]. Despite these gains, these evidences are not sufficient to support or repel the use of consolidative RT in ALNHL. The possible interaction between rutuximab and radiation therapy makes the results of these combinations unpredictable. Thus, radiation therapy should be studied in this new context, and cannot be considered a standard of care until its benefit is proven. Some studies have recently addressed the role of radiation therapy in the management of localized disease in patients with positive Positron Emission Tomography (PET) after chemotherapy [43-45]. Radiation therapy yielded interesting survival results in this subset of patients, providing a rational selection tool for consolidative radiotherapy.

There are still many unanswered questions regarding the management of early stage aggressive NHL. The ideal chemotherapy regimen, its optimal duration, the duration of rituximab treatment, the benefits of adding RT and its dose are important issues that need further evaluation by high-quality RCT. Our systematic review suggests that RT prolongs PFS and can be considered an option for patients who cannot tolerate a high dose or prolonged schedule of chemotherapy. Further evaluation of RT after chemotherapy is still needed.

## Conclusions

In conclusion, RT prolongs PFS, with no impact on OS. It must be considered an option for patients who cannot tolerate a high dose or prolonged schedule of chemotherapy. Further investigation of the role of RT in the era of targeted therapy is needed.

## Competing interests

There are no actual or potential conflicts of interest from the authors.

## Authors’ contributions

Conception and design: LVS and ADS; Acquisition of data: all authors; Analysis and interpretation of data: all authors; Manuscript drafting: LVS, JPL and ADS; Manuscript revising: all authors; final approval of this version: all authors. All Authors read and approved the final manuscript.

## Funding

The authors received no specific funding for this study.
